# High complement activation index predicts renal progression in crescentic IgA nephropathy

**DOI:** 10.3389/fimmu.2026.1915157

**Published:** 2026-09-14

**Authors:** Jingjing Wang, Ling Jiang, Yuhui Luo, Yuanmao Tu, Ju'an Wang, Xinyue Wang, Shaoshan Liang, Ling Wang, Haitao Zhang, Xianghua Huang

**Affiliations:** 1 National Clinical Research Center for Kidney Diseases, Jinling Hospital, Affiliated Hospital of Medical School, Nanjing University, Nanjing, China; 2 Department of Nephrology, 923rd Hospital of the Joint Logistics Support Force, Nanning, China

**Keywords:** complement activation index, crescentic lesions, IgA nephropathy, prognosis, renal outcome

## Abstract

**Background:**

Crescentic immunoglobulin A nephropathy (IgAN) with Oxford C2 lesions (≥25% crescents) represents a severe disease phenotype. Individual complement markers show inconsistent prognostic value. We developed a composite Complement Activation Index (CAI) and tested its association with renal progression.

**Methods:**

This study enrolled 157 biopsy-confirmed patients with crescentic IgAN. The CAI was derived from four components: (i) urinary C3 > 2.8mg/L, (ii) serum C3 < 0.80 g/L, (iii) glomerular C3 immunofluorescence intensity ≥ 2+, and (iv) presence of glomerular C1q deposition. Each component contributed 1 point, categorizing patients into low- CAI and high- CAI groups. The association between CAI and the composite kidney disease progression event, defined as doubling of serum creatinine or end-stage renal disease, was assessed using Cox proportional hazards models.

**Results:**

A high CAI (43.9%, 69/157) was associated with worse baseline renal function (eGFR 39 vs. 65 mL/min/1.73 m², P<0.001), higher proteinuria (4.6 vs. 2.3 g, P<0.001), and more advanced tubulointerstitial injury. Over a median follow-up of 34.1 months, 44 patients (28.0%) reached the composite endpoint. High CAI independently predicted adverse outcomes (adjusted HR 2.58, 95% CI 1.15–5.80, P = 0.022), with each 1-point increase associated with an 87% higher risk (HR 1.87, 95% CI 1.10–3.20, P = 0.021). Adding CAI to the Oxford MEST model improved time-dependent AUC at 36 months (0.79 vs. 0.75, ΔAUC 0.04, 95% CI -0.02 to 0.12).

**Conclusion:**

Higher CAI was independently associated with renal progression in crescentic IgAN. As an exploratory composite index, CAI may complement existing pathological risk stratification, but its incremental clinical utility requires prospective validation.

## 1 Introduction

IgA nephropathy (IgAN) is the most common primary glomerulonephritis worldwide and may carry a substantial risk of progressive kidney function decline (1). Although the clinical course is heterogeneous, the presence of crescentic lesions is associated with an aggressive phenotype. Patients with Oxford classification C2 lesions (crescents in ≥25% of glomeruli) appear to be at particularly high risk, with possible rapid progression to end-stage kidney disease (ESKD) if not promptly treated (2, 3). Improved risk stratification in this subgroup may therefore help guide timely intervention.

The Oxford MEST-C scoring system offers a standardized histopathological assessment and has been shown to predict long-term outcomes (4, 5). However, this system largely captures structural damage and chronicity, with limited ability to quantify active immune pathways. The complement system is increasingly recognized as an important driver of glomerular injury in IgAN, particularly through the alternative and lectin pathways (6–8). Crescent formation has been closely linked to complement activation, and urinary complement split products have been reported to correlate with the proportion of crescents (9, 10). Nevertheless, individual complement markers – such as serum C3, arteriolar C4d, glomerular C3/C5 convertases, or glomerular C4d – have shown inconsistent prognostic value when used alone, possibly due to biological variability and the multifactorial nature of complement consumption (11–14).

To address these limitations, we developed a composite Complement Activation Index (CAI) that combines four routinely available parameters: urinary C3 (above the upper normal limit), serum C3 (below the lower normal limit), intensity of glomerular C3 immunofluorescence (≥2+ on a 0–3+ scale), and presence of glomerular C1q deposition. These components were selected because they may collectively reflect systemic consumption (serum C3), local tissue deposition (glomerular C3), tubular leakage of activated fragments (urinary C3), and classical pathway engagement (C1q). All four parameters were routinely collected at our center. However, urinary C3 measurement is not part of standard testing in most clinical laboratories and is currently restricted to selected centers with validated urinary complement assays. Therefore, the CAI should be considered a proof-of-concept index, and broader implementation would require assay standardization or the identification of more widely available complementary markers. Other pathway-specific markers (e.g., properdin, MBL, MASP-2) were not included because they are not routinely measured in our center, and retrospective data were unavailable (15).

The objective of this study was to evaluate the prognostic value of the CAI in a retrospective cohort of patients with biopsy-proven crescentic IgAN (Oxford C2) and to provide preliminary evidence for the clinical utility of the CAI.

## 2 Methods

### 2.1 Study design and participants

This retrospective cohort study included patients with biopsy-proven IgA nephropathy diagnosed between January 2016 and January 2025. The inclusion criteria were: (i) age ≥ 18 years; (ii) availability of follow-up data for at least 6 months after renal biopsy (patients who experienced the composite endpoint within the first 6 months were retained as long as post-event follow-up records were available; patients who were lost to follow-up within 6 months, including those who initiated renal replacement therapy and did not return for follow-up, were excluded); (iii) availability of complete key baseline clinical, laboratory, and histological data; and (iv) presence of crescentic lesions involving ≥25% of glomeruli (Oxford C2 lesion). Patients with secondary IgA nephropathy (e.g., Henoch-Schönlein purpura, systemic lupus erythematosus, liver cirrhosis) were excluded. During the study period, a total of 5,390 patients underwent renal biopsy and were diagnosed with primary IgA nephropathy at our center. Among these, 181 patients (3.4%) had crescentic lesions involving ≥25% of glomeruli (Oxford C2 lesion). Of these 181 C2 patients, 157 (86.7%) had complete baseline clinical, laboratory, and histopathological data as well as at least 6 months of follow-up and were included in the final analysis. The remaining 24 were excluded due to missing key baseline data (n = 15) or loss to follow-up within 6 months (n = 9). The study was approved by the institutional ethics committee (Approval Number: 2025DZKY-116-01), and the requirement for informed consent was waived given the retrospective nature of the study.

### 2.2 Data collection

Baseline demographic, clinical, and laboratory data were collected at the time of renal biopsy. Demographic data included age and sex. Clinical variables comprised body mass index (BMI), mean arterial pressure (MAP), history of hypertension, diabetes mellitus, tonsillitis, and gross hematuria. Laboratory parameters included 24-hour urine protein (UPRO), urinary C3 (uC3), hemoglobin, platelet count, serum albumin, globulin, creatinine, urea nitrogen, total cholesterol, triglycerides, high-density lipoprotein cholesterol (HDL-C), low-density lipoprotein cholesterol (LDL-C), lactate dehydrogenase (LDH), cystatin C, immunoglobulins (IgG, IgA, IgE, IgM), complement components C3 and C4, and lymphocyte subsets (CD4^+^, CD8^+^, CD20^+^). The estimated glomerular filtration rate (eGFR) was calculated using the CKD-EPI equation.

Peripheral blood lymphocyte subsets, including CD3+CD4+ T cells, CD3+CD8+ T cells, and CD20+ B cells, were analyzed by flow cytometry using fresh whole blood samples. T-cell subsets were stained using the Beckman Coulter CD45/CD4/CD8/CD3 reagent kit (Cat No. 6607013), while CD20+ B cells were assessed in a parallel tube using a CD20 monoclonal antibody (Clone B9E9, Beckman Coulter). Data acquisition was performed on a Cytomics FC 500 flow cytometer (Beckman Coulter), and absolute cell counts were determined using Flow-Count fluorospheres (Beckman Coulter). All procedures followed the routine operating procedures of our clinical flow cytometry laboratory and the guidelines of the International Society for Advancement of Cytometry (ISAC).

Urinary C3 was measured in morning spot urine specimens collected within 24 hours before renal biopsy using an immunoturbidimetric assay (BioSTEC Complement 3 Assay Kit, Chongqing BioSTEC Biotechnology Co., Ltd., China), with calibration traceable to the ERM-DA470k/IFCC standard. The manufacturer’s stated linear range for serum samples was 8.0–600.0 mg/dL. Because the kit was developed for serum samples, its urinary application was laboratory-adapted. Urine samples were concentrated before measurement using 10 kDa molecular-weight cut-off centrifugal filters (Merck Millipore, UFC501096), and results were back-calculated to the original urinary concentration. Internal validation using pooled urine samples showed a mean spike-recovery rate of 95% and an intra-assay coefficient of variation of 6.0%. Urinary C3 values were reported as absolute concentrations (mg/L) without normalization to urinary creatinine.

Histopathological data were obtained from renal biopsy reports. The Oxford classification scores (M, E, S, T) were recorded according to the 2016 revision. Immunofluorescence intensity for C3 was graded semiquantitatively (0–3+), and C1q deposition was recorded as positive (≥1+) or negative. All biopsies were reviewed by two experienced renal pathologists who reached a consensus on each case. The pathologists were not blinded to clinical or other pathological data, as this was a retrospective study based on routine diagnostic reports.

Treatment data included the use of renin-angiotensin system blockers (ARB/ACEI), SGLT2 inhibitors, corticosteroids (oral prednisone and methylprednisolone pulse therapy), targeted-release budesonide (NEFECON), mycophenolate mofetil, cyclophosphamide, rituximab, and other immunosuppressive agents.

### 2.3 Definition of the complement activation index

The CAI was developed to integrate multiple lines of evidence of complement activation. It consists of four components: (i) urinary C3 level > 2.8 mg/L (1 point); (ii) serum C3 level < 0.80 g/L (1 point); (iii) glomerular C3 immunofluorescence intensity ≥ 2+ (1 point); and (iv) glomerular C1q immunofluorescence positivity (1 point). Each component was assigned 1 point, yielding a total score ranging from 0 to 4. For the primary analysis, patients were categorized into Low CAI (0–1 points) and High CAI (≥2 points; observed scores 2–3). This threshold was prespecified before outcome analysis and was not selected using ROC analysis or another outcome-driven optimization method. A score of ≥2 was considered to indicate concurrent abnormalities in at least two distinct complement compartments, whereas a score of 0–1 indicated absent or isolated complement-related abnormalities. The resulting groups were reasonably balanced (Low CAI, n = 88; High CAI, n = 69), facilitating between-group comparisons. To avoid dependence on a single dichotomization, CAI was additionally analyzed as a continuous variable, and an alternative cutoff was examined in sensitivity analysis.

The thresholds for urinary C3 (>2.8 mg/L) and serum C3 (<0.80 g/L) were based on the routine clinical reference ranges of our institution, which were established using standard chemiluminescence and turbidimetric assays in a local healthy population. These thresholds have been consistently used in clinical practice for abnormality classification. Glomerular C3 immunofluorescence intensity was graded semi-quantitatively by an experienced renal pathologist (0–3+, with ≥2+ considered positive). C1q positivity was defined as any detectable glomerular deposition (≥1+).

### 2.4 Outcome definition

The primary outcome was a composite renal endpoint, defined as doubling of serum creatinine from baseline or progression to end-stage kidney disease (ESKD), which was defined as the initiation of hemodialysis, peritoneal dialysis, renal transplantation, or an eGFR of less than 15 ml/min/1.73m^^2^ for 3 months. The follow-up time was calculated from the date of renal biopsy to the date of the endpoint event or the last available follow-up visit.

### 2.5 Statistical analysis

Continuous variables are expressed as mean ± standard deviation (SD) or median with interquartile range (IQR), depending on their distribution. Categorical variables are presented as frequencies and percentages. Baseline characteristics were compared between the Low CAI and High CAI groups using the Mann-Whitney U test for continuous variables, and the chi-squared test or Fisher’s exact test for categorical variables, as appropriate.

For baseline characteristic comparisons presented in **Tables 1**, **2**, nominal P values were calculated for descriptive purposes. To account for multiplicity, Benjamini–Hochberg false discovery rate correction was applied across all 49 baseline comparisons. Both nominal P values and FDR-adjusted Q values are provided in **Supplementary Table 3**. Associations with Q < 0.05 were considered statistically significant after multiple-testing correction, whereas associations with nominal P < 0.05 but Q ≥ 0.05 were interpreted as exploratory.

**Table 1:** Baseline demographic, clinical, and laboratory characteristics stratified by complement activation index (CAI).

Variable	Total (N = 157)	Low CAI (Score 0–1) (n=88)	High CAI (Score 2–3) (n=69)	P
Demographics				
Age, years	40 ± 14	41 ± 14	40 ± 14	0.486
Female, n (%)	96 (61.1)	54 (61.4)	42 (60.9)	1.000
BMI, kg/m²	24.4 ± 3.7	24.2 ± 3.7	24.7 ± 3.7	0.374
MAP, mmHg	98 ± 12	97 ± 12	100 ± 11	0.112
Co-morbidities				
Hypertension, n (%)	65 (41.4)	31 (35.2)	34 (49.3)	0.083
Diabetes mellitus, n (%)	6 (3.8)	5 (5.7)	1 (1.5)	0.229
Laboratory data				
UPRO, g/24h	2.7 (1.7-5.2)	2.3 (1.4–3.5)	4.6 (2.2–7.5)	**<0.001**
Urinary C3, mg/L	3.0 (2.0-10.0)	2.0 (2.0-3.1)	7.4 (3.6-25.4)	**<0.001**
Hemoglobin, g/L	115 ± 22	116 ± 19	113 ± 26	0.154
Platelets, ×10^9^/L	253 ± 86	264 ± 91	238 ± 78	0.073
Albumin, g/L	35.3 ± 6.0	37.2 ± 5.0	32.8 ± 6.3	**<0.001**
Globulin, g/L	22.0 ± 4.4	22.8 ± 3.9	21.1 ± 4.8	**0.010**
Serum creatinine, mg/dL	1.4 (0.9-2.2)	1.2 (0.9-1.8)	1.8 (1.2-2.8)	**<0.001**
eGFR, mL/min/1.73m²	55 (29-81)	65(38-92)	39 (26-64)	**<0.001**
Urea nitrogen, mg/dL	25.1 ± 13.6	21.9 ± 11.5	29.0 ± 15.1	**0.001**
Total cholesterol, mmol/L	5.7 ± 1.7	5.3 ± 1.5	6.2 ± 1.8	**0.003**
Triglycerides, mmol/L	1.6 (1.2-2.6)	1.4 (1.1-2.1)	2.0 (1.5-2.8)	**0.003**
HDL-C, mmol/L‡	1.2 ± 0.4	1.1 ± 0.4	1.3 ± 0.5	**0.078**
LDL-C, mmol/L‡	3.2 ± 1.3	3.0 ± 1.2	3.5 ± 1.4	**0.036**
LDH, U/L†	207 ± 65	191 ± 41	228 ± 82	**0.008**
Cystatin C, mg/L†	1.6 ± 0.8	1.3 ± 0.6	1.9 ± 0.8	**<0.001**
IgG, g/L§	9.3 ± 3.6	10.1 ± 3.1	8.2 ± 3.8	**<0.001**
IgE, IU/mL§	45 (22-105)	51 (20-111)	43 (25-90)	0.995
IgA, g/L§	3.0 ± 1.1	2.9 ± 1.1	3.0 ± 1.2	0.925
IgM, g/L§	1.1 ± 0.5	1.1 ± 0.6	1.0 ± 0.5	0.149
Complement C3, g/L	1.00 ± 0.22	1.04 ± 0.18	0.94 ± 0.24	**0.001**
Complement C4, g/L	0.27 ± 0.09	0.27 ± 0.09	0.26 ± 0.09	0.489
CD4+ T cells,/μL‖	780 ± 377	809 ± 383	745 ± 369	0.394
CD8+ T cells,/μL‖	433 ± 215	443 ± 215	421 ± 215	0.487
CD20+ cells,/μL¶	238 ± 141	262 ± 152	211 ± 125	**0.046**

Data are presented as mean ± SD (median, Q1–Q3) for continuous variables and n (%) for categorical variables.

P-values: continuous variables were compared with the Mann-Whitney U test; categorical variables with the chi-square test or Fisher’s exact test as appropriate.

‡ HDL-C and LDL-C data were available in 144 total patients (Low CAI: 83; High CAI: 61).

† LDH and Cystatin C were available in 139 and 140 total patients, respectively (Low CAI: 78/79; High CAI: 61/61).

§ Immunoglobulins available in 148–152 total patients (Low CAI: 81–84; High CAI: 67–68).

‖ CD4/CD8 available in 135 total patients (Low CAI: 73; High CAI: 62).

¶ CD20 available in 130 total patients (Low CAI: 69; High CAI: 61). BMI, body mass index; MAP, mean arterial pressure; UPRO, 24-hour urine protein; eGFR, estimated glomerular filtration rate; HDL-C, high-density lipoprotein cholesterol; LDL-C, low-density lipoprotein cholesterol; LDH, lactate dehydrogenase.

Nominal P values are shown. FDR-adjusted Q values for all baseline comparisons are provided in **Supplementary Table 3**. Associations with nominal P < 0.05 but Q ≥ 0.05 should be interpreted as exploratory.

Continuous variables are reported with a level of precision consistent with routine laboratory reporting and clinical interpretability. P values ≥0.001 are reported to three decimal places, and P values <0.001 are reported as P < 0.001.

**Table 2:** Pathological characteristics and treatment regimens stratified by complement activation index (CAI).

Variable	Total (n=157)	Low CAI (Score 0–1) (n=88)	High CAI (Score 2–3) (n=69)	P
Oxford (MEST) classification, n (%)				
M1	55 (35.0)	24 (27.3)	31 (44.9)	**0.026**
E1	99 (63.1)	56 (63.6)	43 (62.3)	0.865
S1	152 (96.8)	84 (95.5)	68 (98.6)	0.401
T category				**0.009**
T0	119 (75.8)	75 (85.2)	44 (63.8)	
T1	31 (19.7)	11 (12.5)	20 (29.0)	
T2	7 (4.5)	2 (2.3)	5 (7.3)	
Immunofluorescence, n (%)				
IgG deposition				0.899
0	128 (81.5)	73 (83.0)	55 (79.7)	
1+	22 (14.0)	11 (12.5)	11 (15.9)	
2+	7 (4.5)	4 (4.6)	3 (4.4)	
IgA deposition				0.774
1+	3 (1.9)	2 (2.3)	1 (1.5)	
2+	118 (75.2)	64 (72.7)	54 (78.3)	
3+	36 (22.9)	22 (25.0)	14 (20.3)	
IgM deposition				0.116
0	96 (61.2)	59 (67.1)	37 (53.6)	
1+	52 (33.1)	23 (26.1)	29 (42.0)	
2+	9 (5.7)	6 (6.8)	3 (4.4)	
C3 deposition				**<0.001**
0	20 (12.7)	19 (21.6)	1 (1.5)	
1+	31 (19.8)	27 (30.7)	4 (5.8)	
2+	92 (58.6)	36 (40.9)	56 (81.2)	
3+	14 (8.9)	6 (6.8)	8 (11.6)	
C1q positive (≥1+)	3 (1.9)	0 (0.0)	3 (4.3)	0.081
Treatment, n (%)				
Baseline CRRT	3 (1.9)	2 (2.3)	1 (1.5)	1.000
Medications, n (%)				
ARB/ACEI	99 (63.1)	62 (70.5)	37 (53.6)	**0.029**
SGLT2 inhibitor	18 (11.5)	14 (15.9)	4 (5.8)	0.081
Methylprednisolone pulse	88 (56.1)	44 (50.0)	44 (63.8)	0.106
Prednisone	143 (91.1)	80 (90.9)	63 (91.3)	1.000
NEFECON	10 (6.4)	6 (6.8)	4 (5.8)	1.000
Mycophenolate mofetil	56 (35.7)	32 (36.4)	24 (34.8)	0.870
Cyclophosphamide	14 (8.9)	5 (5.7)	9 (13.0)	0.151
Rituximab	3 (1.9)	2 (2.3)	1 (1.5)	1.000
Other immunosuppressant	11 (7.0)	6 (6.8)	5 (7.3)	1.000

P values: categorical variables compared with the chi-square test or Fisher’s exact test as appropriate. ARB/ACEI, angiotensin receptor blocker/angiotensin-converting enzyme inhibitor; SGLT2, sodium-glucose cotransporter 2; NEFECON, targeted-release budesonide; CRRT, continuous renal replacement therapy.

Nominal P values are shown. FDR-adjusted Q values for all baseline comparisons are provided in **Supplementary Table 3**. The differences in M1 lesions and ARB/ACEI use were nominally significant but did not remain significant after Benjamini–Hochberg correction.

Spearman correlation coefficients were computed to explore the relationships between CAI and other clinicopathological variables. A heatmap was generated to visualize the correlation matrix; significant correlations were indicated with asterisks.

Renal survival was estimated using the Kaplan-Meier method, and groups were compared with the log-rank test. Unadjusted and adjusted hazard ratios (HRs) with 95% confidence intervals (CIs) were derived from Cox proportional hazards models. The proportional hazards assumption was tested using Schoenfeld residuals. Three adjustment levels were employed: Model 1, unadjusted; Model 2 (Adjust I), adjusted for age, sex, history of hypertension, and diabetes mellitus; Model 3 (Adjust II), additionally adjusted for baseline eGFR, 24-hour urine protein, MEST scores, and immunosuppressive therapy. CAI was also modeled as a continuous variable (per 1-point increase) to test for a dose-response trend.

For the Cox regression analyses, Models 1–3 represented sequential covariate adjustment for the same underlying association between CAI and the composite renal outcome, rather than independent hypothesis tests. Primary inference was based on the fully adjusted model (Model 3, Adjust II). P values for the unadjusted and partially adjusted models are presented as nominal values to demonstrate the stability of the association. Continuous CAI was analyzed as a prespecified secondary assessment of dose–response.

Several sensitivity analyses were performed to evaluate the robustness of the primary findings: (i) landmark analysis excluding events occurring within the first 6 months after biopsy; (ii) a stratified Cox model with sex as the stratification variable to address a mild departure from the proportional hazards assumption; (iii) an alternative CAI cutoff (0–2 vs. 3-4); and (iv) a model additionally adjusting for the individual components of CAI.

All semiquantitative histopathological and immunofluorescence variables were treated as categorical variables. Oxford M, E, and S scores were entered as binary variables (0 vs. 1), and the T score was entered as a three-level categorical factor with T0 as the reference category. Glomerular C3 immunofluorescence intensity was analyzed according to its semiquantitative staining grades (0, 1+, 2+, and 3+), and C1q deposition was analyzed as a binary variable (negative vs. positive). None of these semiquantitative variables were entered into the regression models as continuous parameters.

The incremental prognostic value of CAI beyond histologic classification was assessed using time-dependent receiver operating characteristic (ROC) curves. The linear predictor of a Cox model containing M, E, S, and T scores was used as the baseline predictor, and the area under the curve (AUC) of the model with and without the continuous CAI score was compared. Time-dependent AUCs at 36 and 60 months were calculated using the Kaplan-Meier method. Bootstrap resampling (1,000 iterations) was performed to obtain 95% confidence intervals for the AUCs and for the difference in AUCs (ΔAUC).

All statistical tests were two-sided, and a P value < 0.05 was considered statistically significant. Analyses were performed using R software, version 4.5.2.

## 3 Results

### 3.1 Study population and baseline characteristics

A total of 157 patients with biopsy-proven crescentic IgAN were enrolled. During the study period, 5,390 patients were diagnosed with IgA nephropathy at our center, of whom 181 (3.4%) met the Oxford C2 criterion. Among these 181 C2 patients, 157 (86.7%) had complete baseline and follow-up data and were included in the final analysis; the remaining 24 were excluded due to missing data or loss to follow-up. Mean age was 40 ± 14 years, and 96 (61.1%) were female. Median eGFR was 55 mL/min/1.73 m2 (IQR 29-81), and median 24-hour proteinuria was 2.7 g (IQR 1.7-5.2). Mean serum C3 was 1.00 ± 0.22 g/L. Oxford MEST distribution: M1 35.0%, E1 63.1%, S1 96.8%, T1 19.7%, T2 4.5%. Baseline immunosuppression included prednisone (91.1%), methylprednisolone pulse (56.1%), mycophenolate mofetil (35.7%), and cyclophosphamide (8.9%). Renin-angiotensin system blockers were used in 63.1%, and SGLT2 inhibitors in 11.5%. Detailed baseline data are shown in **Tables 1**, **2**.

### 3.2 Distribution of CAI scores

The theoretical range of the CAI is 0 to 4 points. In our cohort, the observed scores were distributed as follows: 0 points (n=18, 11.5%), 1 point (n=70, 44.6%), 2 points (n=60, 38.2%), and 3 points (n=9, 5.7%). No patient achieved a score of 4 points. This reflects the fact that among the three patients with C1q positivity, none simultaneously met all three remaining criteria (urinary C3 >2.8 mg/L, serum C3 <0.80 g/L, and glomerular C3 ≥2+). Thus, the maximum observed complement burden in this crescentic IgAN cohort was 3 out of 4 possible components.

### 3.3 Clinicopathological features by complement activation index

All clinical, laboratory, and histopathological parameters described below were assessed at the time of renal biopsy (baseline).Compared with the Low-CAI group, the High-CAI group had worse baseline renal function, including higher serum creatinine, lower eGFR, higher cystatin C, and higher urea nitrogen, all of which remained significant after FDR correction. Baseline proteinuria was higher and serum albumin lower in the High-CAI group. Total cholesterol and triglycerides also remained significantly higher after FDR correction, whereas the difference in LDL-C was only nominally significant and did not survive FDR correction (P = 0.036, Q = 0.098).

Regarding complement and immune parameters, serum C3 was lower, urinary C3 was higher, and serum IgG was lower in the High-CAI group, all of which remained significant after FDR correction. The difference in circulating CD20+ B-cell counts was nominally significant but did not remain significant after FDR correction (P = 0.046, Q = 0.120) (**Table 1**; **Supplementary Table 3**).

On renal histology (evaluated at baseline biopsy), the High-CAI group had more advanced tubulointerstitial damage, reflected by higher Oxford T scores, and this difference remained significant after FDR correction (P = 0.009, Q = 0.031). M1 lesions were more frequent in the High-CAI group at the nominal level, but this association did not survive FDR correction (P = 0.026, Q = 0.080). E and S scores were comparable between the groups. Glomerular C3 deposition remained stronger in the High-CAI group after FDR correction (P < 0.001, Q = 0.003), whereas the difference in C1q deposition was not statistically significant.

At baseline, ARB/ACEI use was less frequent in the High-CAI group at the nominal level, but this difference did not remain significant after FDR correction (P = 0.029, Q = 0.084). SGLT2 inhibitor use did not differ significantly between the groups (P = 0.081). Baseline corticosteroid and other immunosuppressive treatment patterns were also comparable (**Table 2**; **Supplementary Table 3**).

### 3.4 Correlations between CAI and clinicopathological parameters

Spearman correlation analysis showed that CAI was positively correlated with 24-hour urine protein (r = 0.376, P < 0.001) and negatively correlated with eGFR (r = –0.390, P < 0.001). Among the Oxford MEST components, CAI was modestly but significantly associated with T score (r = 0.235, P = 0.003) and M score (r = 0.177, P = 0.027). Correlations with age, sex, BMI, MAP, E score, and S score were not significant (**Figure 1**; **Supplementary Table 1**).

**Figure 1:**
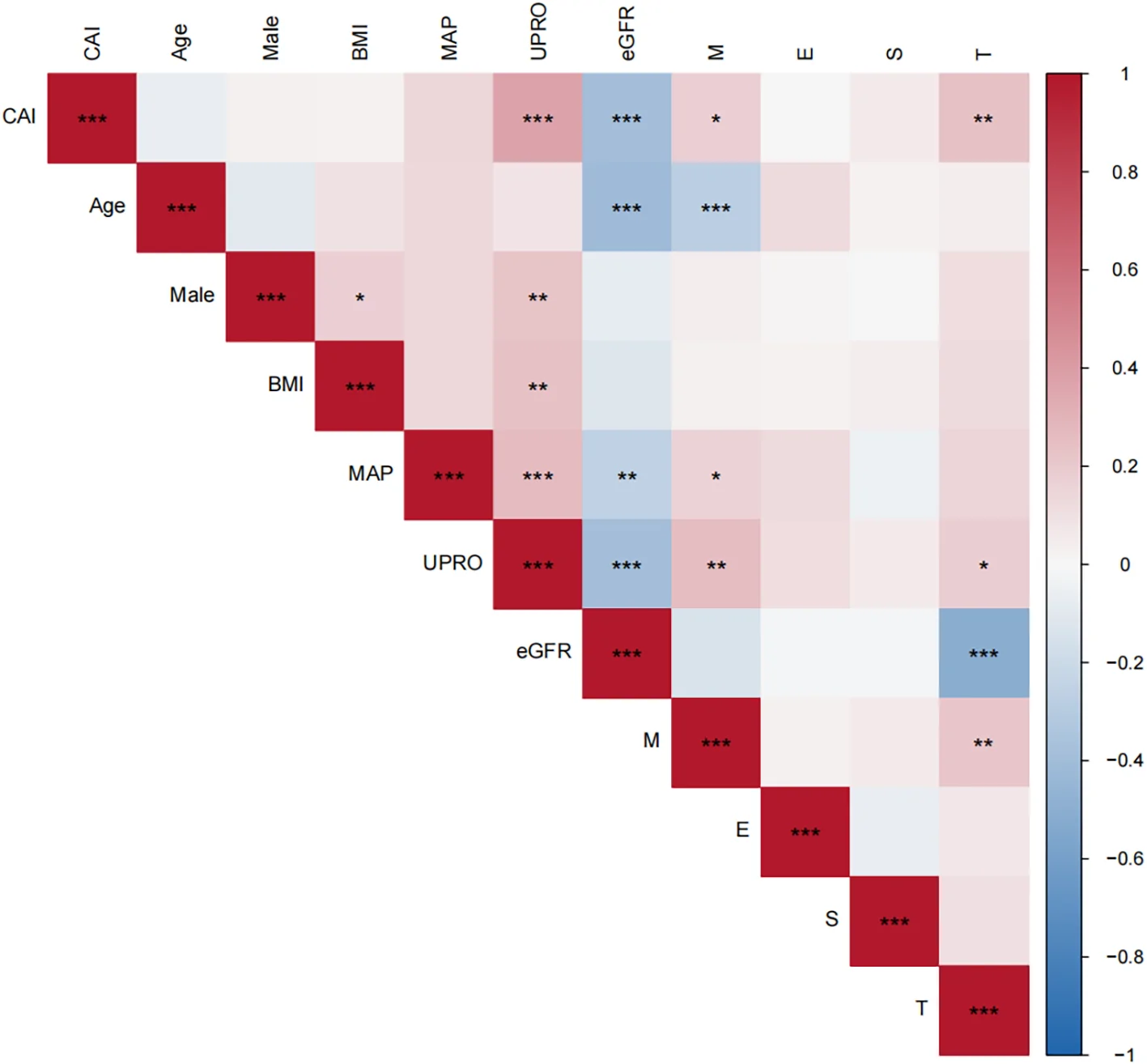
The heatmap shows correlations between parameters relevant for IgAN in the total group. The color scale ranges from blue (negative correlation) to red (positive correlation), with white indicating a correlation of zero. The intensity of the color is proportional to the absolute correlation coefficient (r). Significant correlations are marked with asterisks: *P < 0.05, **P < 0.01, ***P < 0.001. CAI, complement activation index; BMI, body mass index; MAP, mean arterial pressure; UPRO, 24-hour urine protein; eGFR, estimated glomerular filtration rate; M, mesangial hypercellularity; E, endocapillary proliferation; S, segmental sclerosis; T, tubular atrophy/interstitial fibrosis.

### 3.5 Association of CAI with the composite renal outcome

Over a median follow-up of 34.1 months (IQR 12.6–75.1), 44 patients (28.0%) reached the composite endpoint. Kaplan–Meier analysis demonstrated significantly worse renal survival in the High CAI group compared with the Low CAI group (log-rank P < 0.001) (**Figure 2**).

**Figure 2:**
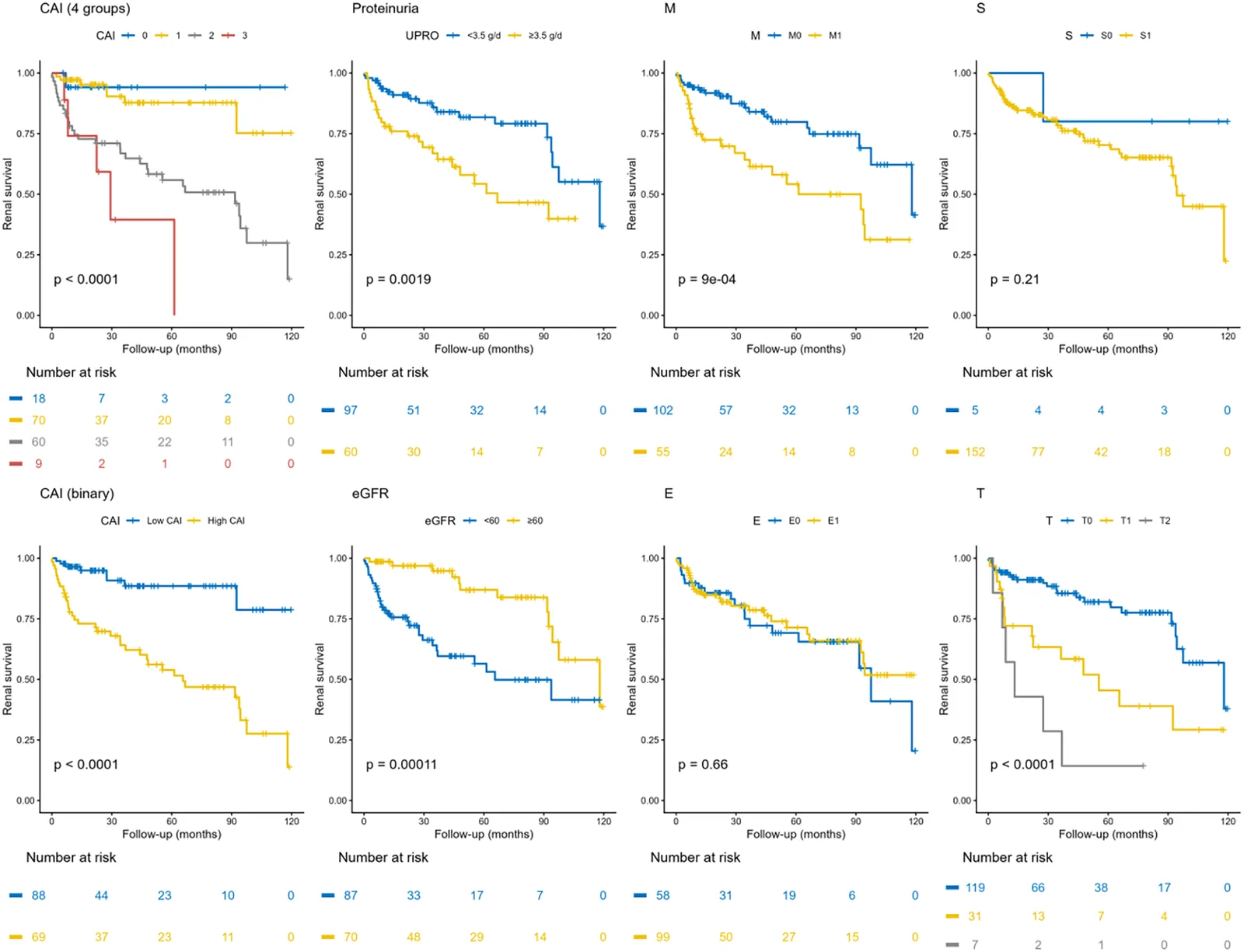
Kaplan-Meier estimates of renal survival according to different groups.

In unadjusted Cox proportional hazards regression, High CAI (vs. Low CAI) conferred a 5.42-fold increased risk of the composite outcome (95% CI 2.51–11.68, P < 0.001). After adjustment for age, sex, hypertension, and diabetes mellitus (Adjust I), the hazard ratio remained statistically significant at 4.57 (95% CI 2.10–9.96, P < 0.001). With further adjustment for eGFR, proteinuria, Oxford MEST scores, and immunosuppressive therapy (Adjust II), the association persisted (HR 2.58, 95% CI 1.15–5.80, P = 0.022). When CAI was analyzed as a continuous variable, each 1-point increase was independently associated with an 87% higher risk of the endpoint (HR 1.87, 95% CI 1.10–3.20, P = 0.021) (**Table 3**).

**Table 3:** Association between complement activation index (CAI) and the composite renal outcome.

Exposure	Non-adjusted		Adjust I		Adjust II	
	HR (95% CI)	P Value	HR (95% CI)	P Value	HR (95% CI)	P Value
CAI (per 1 point increase)	3.07 (1.94–4.88)	<0.001	2.63 (1.64–4.22)	<0.001	1.87 (1.10–3.20)	0.021
Low CAI (Score 0–1)	1.0 (Reference)		1.0 (Reference)		1.0 (Reference)	
High CAI (Score 2–3)	5.42 (2.51–11.68)	<0.001	4.57 (2.10–9.96)	<0.001	2.58 (1.15–5.80)	0.022

Non-adjusted model: Adjusted for none.

Adjusted I model: Adjusted for gender, age, hypertension history, and diabetes mellitus history.

Adjusted II model: Adjusted for gender, age, hypertension history, diabetes mellitus history, eGFR, UPRO, M, E, S, T, and immunosuppressive regimen.

P values are nominal. Models 1–3 represent sequential adjustment for the same underlying exposure-outcome association rather than independent hypothesis tests. Primary inference was based on the High versus Low CAI comparison in the fully adjusted model. Continuous CAI was a prespecified secondary analysis of dose-response.

### 3.6 Sensitivity analyses

A series of sensitivity analyses confirmed the robustness of the primary findings. The association between High CAI and the composite outcome remained significant after excluding events within the first 6 months (landmark analysis: HR 3.40, 95% CI 1.33–8.67, P = 0.010) and after stratifying by sex to address mild violation of the proportional hazards assumption (HR 3.36, 95% CI 1.48–7.63, P = 0.004). Additional adjustment for the individual components of CAI (urinary C3, serum C3, immunofluorescence C3, and C1q) alongside clinical covariates yielded a similar point estimate (HR 2.12, 95% CI 0.89–5.05), although the confidence interval was wider and crossed the null (P = 0.090), suggesting that the prognostic value of CAI may be largely explained by its individual components, although the wide confidence interval indicates imprecision. By contrast, an alternative CAI cutoff (score 0–2 vs. 3–4) did not show a significant association (HR 1.49, 95% CI 0.65–3.42, P = 0.349), supporting the discriminatory value of the original binary definition (0–1 vs. 2–3) (**Supplementary Table 2**).

### 3.7 Incremental prognostic value of CAI

Time-dependent ROC analysis was performed to evaluate whether CAI improves risk discrimination beyond traditional pathological indices (**Figure 3**). The MEST model alone achieved 36-month and 60-month AUCs of 0.75 (95% CI 0.65–0.87) and 0.77 (95% CI 0.67–0.87), respectively. Adding CAI to the MEST model increased the AUC to 0.79 (95% CI 0.72–0.89) at 36 months and to 0.79 (95% CI 0.73–0.88) at 60 months, corresponding to absolute increments of 0.04 (95% CI –0.02 to 0.12) and 0.02 (95% CI –0.03 to 0.10) (**Table 4**).

**Figure 3:**
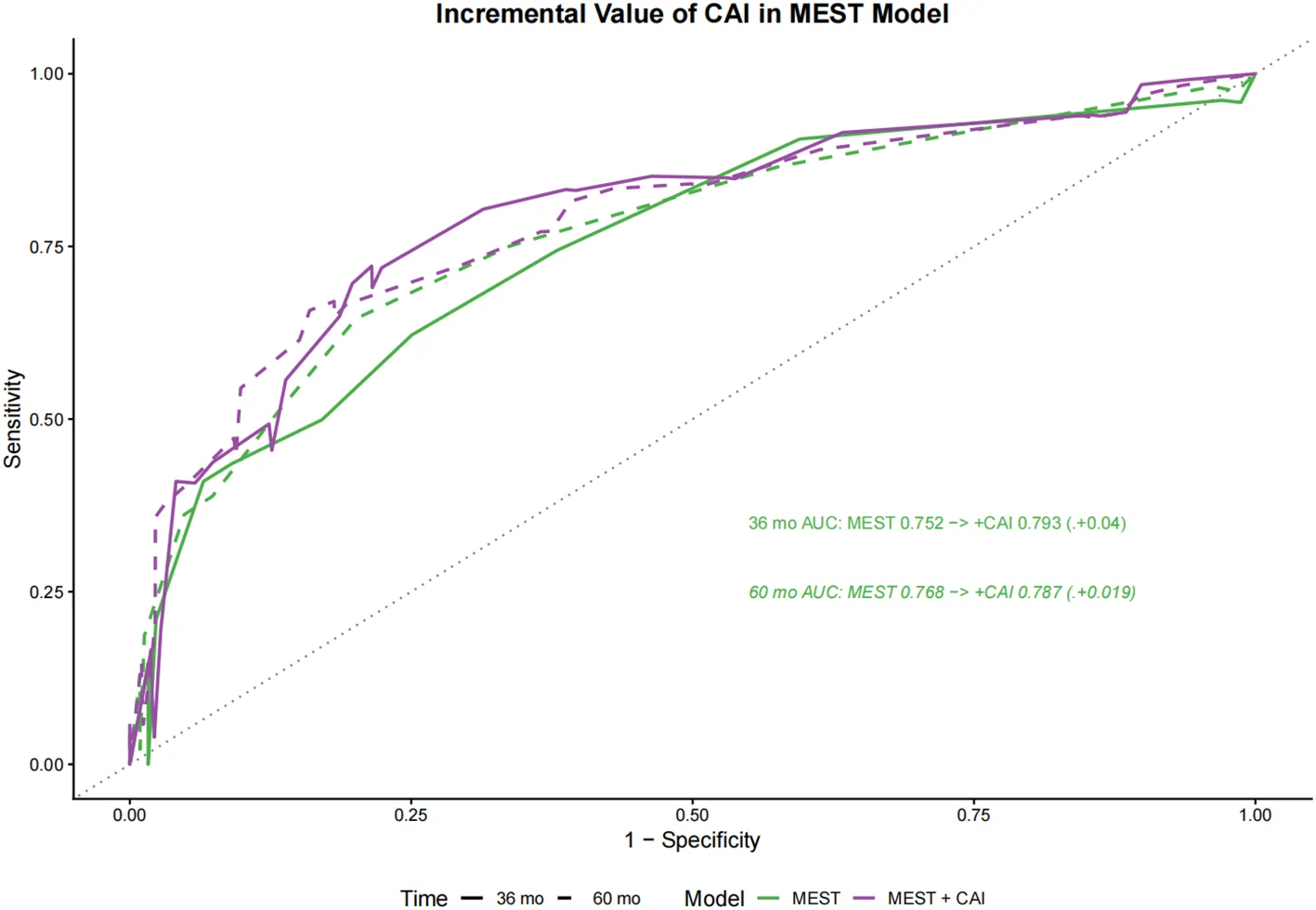
Time-dependent receiver operating characteristic (ROC) curves for the prediction of 3-year and 5-year renal survival.

**Table 4:** Time-dependent area under the receiver operating characteristic curve (AUC) for predicting renal survival at 36 and 60 months, with 95% bootstrap confidence intervals.

Model	36-month AUC (95% CI)	60-month AUC (95% CI)
CAI	0.70 (0.61–0.79)	0.69 (0.61–0.76)
MEST	0.75 (0.65–0.87)	0.77 (0.67–0.87)
CAI + MEST	0.79 (0.72–0.89)	0.79 (0.73–0.88)
Δ (CAI+MEST − MEST)	0.04 (-0.02–0.12)	0.02 (-0.03–0.10)

AUC values were estimated using the Kaplan–Meier method. 95% confidence intervals (CIs) were derived from 1,000 bootstrap resamples with the percentile method. Δ denotes the absolute change in AUC when CAI is added to the MEST model. The composite renal outcome was defined as doubling of serum creatinine or end-stage kidney disease.Abbreviations: CAI, complement activation index; MEST, Oxford classification; eGFR, estimated glomerular filtration rate.

## 4 Discussion

Crescentic IgA nephropathy (IgAN), characterized by extracapillary proliferation involving ≥25% of glomeruli, represents a distinct high-risk phenotype with an ominous prognosis (16, 17). Although the Oxford MEST-C scoring system provides a standardized framework for pathological evaluation, it largely reflects structural damage and may not fully capture the dynamic activation of immune pathways, in particular complement activation implicated in crescent formation. Current therapeutic strategies, which rely largely on broad immunosuppression, show variable efficacy (18, 19). This suggests a need for biomarkers that may better quantify complement-mediated injury to aid risk stratification and guide more individualized management in this population.

To address this gap, we developed the Complement Activation Index (CAI), a composite score integrating urinary C3, serum C3, glomerular C3 immunofluorescence, and C1q deposition. In our cohort, higher CAI scores were associated with more severe baseline renal impairment, heavier proteinuria, and greater histological damage. After adjustment for established clinical and pathological variables, high CAI status remained associated with an increased risk of the composite renal outcome (adjusted HR 2.58; 95% CI 1.15–5.80). However, the addition of CAI to the Oxford MEST model yielded a modest increase in time-dependent AUC (ΔAUC 0.04 at 36 months), which was not statistically significant (95% CI -0.02 to 0.12). This modest increment may partly reflect measurement variability in semiquantitative MEST and immunofluorescence scoring. Accordingly, the present findings support CAI as a candidate, biologically interpretable index of complement activation burden, but do not establish a statistically significant improvement in model discrimination beyond MEST. The limited sample size (n = 157) and number of outcome events (n = 44) likely contributed to the wide confidence interval and preclude a definitive conclusion regarding the incremental predictive value of CAI.

In our crescentic IgAN cohort, baseline renal impairment was severe, and segmental sclerosis (S1 lesions) was nearly universal (96.8%), suggesting that chronic structural damage was already present at the time of biopsy, consistent with recent proteomic studies linking extracellular matrix accumulation to progressive IgAN (21). Furthermore, the high CAI group had higher proteinuria, lower eGFR, a nominally higher frequency of M1 lesions, and greater chronic tubulointerstitial damage (higher T scores). These findings reflect a disease state where active complement-driven inflammation coexists with established chronic structural injury. It is important to note that in the Oxford classification, T lesions represent chronic tubulointerstitial scarring, whereas cellular and fibrocellular crescents (C lesions) and endocapillary hypercellularity (E lesions) are the true markers of active injury. Local and systemic complement dysregulation may therefore contribute to the progression of mesangial proliferation and tubulointerstitial fibrosis in crescentic IgAN.

Deposition of terminal complement complexes, such as C5b-9 (membrane attack complex, MAC), has been implicated in glomerular and tubulointerstitial injury in IgAN. In glomeruli, C5b-9 deposition correlates positively with the extent of focal glomerulosclerosis, tubular atrophy, and interstitial inflammation, and is inversely correlated with podocyte number, suggesting that podocyte injury mediated by complement activation may contribute to proteinuria and disease progression (22, 23). Moreover, urinary MAC levels correlate with interstitial fibrosis and the percentage of global glomerular sclerosis, and animal models have shown that C5b-9 formation in tubules can exacerbate tubulointerstitial damage (24–26). In the present study, the concurrent reduction in IgG and CD20+ B-cell counts in the high CAI group might reflect enhanced immune complex clearance or altered B-cell dynamics secondary to intense complement activation. These observations raise the possibility that the CAI may serve not only as a composite marker of complement exposure but also as an indicator of downstream complement-mediated tissue injury.

The independent prognostic value of the CAI, which persisted after adjustment for baseline eGFR, proteinuria, and MEST scores, indicates that complement activation status provides prognostic information beyond traditional hemodynamic or structural risk factors. Whether complement activation represents a distinct pathogenic process cannot be established from this observational study. This observation is consistent with emerging data indicating that single-nephron hyperfiltration can accelerate function decline (27). However, whether complement activation adds prognostic information beyond hemodynamic changes remains speculative. Furthermore, the fact that CAI remained predictive in patients receiving standard immunosuppressive therapy indicates that complement activation status carries prognostic information independent of the treatment regimen recorded at baseline. This should not be interpreted as evidence that corticosteroid-based regimens fail to suppress complement activation, as treatment response, adherence, and changes in therapy over time were not systematically assessed in this retrospective cohort. Complement inhibitors have shown promise in IgAN (17, 20, 28), but our observational data do not permit conclusions about the relative efficacy of corticosteroids versus complement-targeted therapy. Machine learning models have improved prognostic accuracy by integrating diverse clinical variables (29). Whether a biologically grounded index such as the CAI offers advantages in selecting candidates for anti-complement therapy remains to be tested in prospective trials. The dose-dependent increase in risk with each incremental point of the CAI score is consistent with an increasing burden of markers related to complement activation, although a causal relationship cannot be established from observational data. Taken together, these findings suggest that the CAI may help stratify a high-risk subset of patients with crescentic IgAN for whom complement-targeted interventions might be hypothesized to be beneficial; this hypothesis warrants testing in randomized prospective trials.

Several complement-based scoring systems have been proposed in IgAN, and comparing the CAI with these approaches may help to contextualize its potential value. For example, a study of 1191 IgAN patients reported that glomerular C3 and C1q deposits affected the clinicopathologic features of IgAN patients and emerged as independent predictors and risk factors for renal outcomes, with the predictive ability of C3 being slightly better than that of C1q (30). The CAI shares the use of glomerular C3 with that score, but differs in three ways: it combines glomerular C3 with serum C3 (reflecting both local deposition and systemic consumption), incorporates urinary C3, which may indicate ongoing complement leakage and tubular injury, and includes C1q (albeit in a small subset of patients). Similarly, a lower serum C3/C4 ratio has been associated with worse renal outcomes (31), whereas the CAI adds tissue-based and urinary evidence of complement activation. The serum IgA/C3 ratio, particularly when combined with glomerular C3 staining, has also been shown to predict disease severity (32); however, the CAI does not rely on IgA levels (which can be influenced by non-complement factors) and instead focuses directly on complement activation products such as C3 and C1q. A potential advantage of the CAI is the inclusion of urinary C3. Unlike tissue markers that reflect complement deposition at a single time point (the biopsy), urinary C3 levels may change over time in response to disease activity or treatment. This suggests that the CAI could serve as a repeatable, non-invasive tool for monitoring complement activation.

Several limitations of this study should be considered. First, because this was a single-center, biopsy-based cohort from a tertiary referral center, the proportion and clinical characteristics of patients with C2 lesions may not be fully representative of the broader IgAN population. The retrospective design and moderate sample size (n=157) may have limited statistical power, and the improvement in time-dependent AUC when adding CAI to the MEST model was not statistically significant (ΔAUC 0.04 at 36 months; 95% CI −0.02 to 0.12). Accordingly, the CAI should be regarded as an exploratory index rather than a validated clinical prediction tool. Its incremental prognostic and clinical utility should be evaluated in larger prospective cohorts with external validation, including assessment of calibration, reclassification, and decision-analytic benefit, before routine implementation. This also limits generalizability to broader IgAN populations, including those with different ethnic backgrounds or lower crescent proportions. Second, the CAI thresholds (e.g., urinary C3 >2.8 mg/L) were derived from our cohort without external validation, and overfitting cannot be excluded. Third, immunofluorescence scoring for glomerular C3 and C1q was performed by two pathologists in consensus but without blinding to clinical data, and inter-observer agreement (kappa) was not formally assessed, which may have introduced some subjectivity. We also acknowledge that urinary C3 values were not normalized to urinary creatinine. As spot urine samples were used, creatinine normalization could have reduced hydration-related variability. Future prospective studies should consider employing creatinine-normalized urinary C3 or 24-hour urine collections to enhance reproducibility. Finally, the equal weighting of CAI components, while pragmatic for this proof-of-concept study, may not reflect the true biological contribution of each marker. C1q positivity was rare in our cohort (1.9%), meaning the CAI was driven largely by urinary and glomerular C3. In addition, tissue C5b-9 and C4d staining and urinary soluble C5b-9 measurements were unavailable because no residual biopsy tissue or stored urine samples remained for retrospective analysis. These markers may provide complementary information on terminal and lectin pathway activation and should be incorporated into future prospective studies. Future prospective studies in larger cohorts should explore whether differential weighting or replacement of C1q with more prevalent markers (e.g., glomerular C4d or urinary soluble C5b-9) improves prognostic performance and generalizability (33).

In summary, this study introduces the Complement Activation Index (CAI), a composite score integrating serum C3, urinary C3, glomerular C3, and C1q. A high CAI (2–3 points) was associated with a 2.6-fold increased risk of kidney function decline or ESKD in patients with crescentic IgAN after adjustment for conventional risk factors and Oxford MEST scores. The consistency of this association across sensitivity analyses and the observed dose-response effect (87% risk increase per CAI point) is consistent with the biological relevance of integrated complement assessment. Given the retrospective design and the lack of statistically significant incremental discrimination beyond MEST, the CAI should be viewed as an exploratory prognostic index requiring external and prospective validation.

## Data Availability

The raw data supporting the conclusions of this article will be made available by the authors, without undue reservation.
