# Supplementary material for: High complement activation index predicts renal progression in crescentic IgA nephropathy
Source: Front Immunol. 2026 Sep 14;17:1915157. doi: 10.3389/fimmu.2026.1915157 (PMC13617021; doi:10.3389/fimmu.2026.1915157)
Supplement: Supplementary file 1 [file Table1.docx]

Supplementary Material

### Supplementary Table S1. Spearman correlation coefficients between complement activation index (CAI) and baseline clinical-pathological variables.

| **Variable** | **r** | **P value** |
| --- | --- | --- |
| **Age** | −0.062 | 0.442 |
| **Male sex** | 0.034 | 0.675 |
| **BMI** | 0.029 | 0.720 |
| **MAP** | 0.144 | 0.073 |
| **UPRO** | 0.376 | <0.001 |
| **eGFR** | −0.390 | <0.001 |
| **M score** | 0.177 | 0.027 |
| **E score** | 0.009 | 0.913 |
| **S score** | 0.054 | 0.501 |
| **T score** | 0.235 | 0.003 |

Abbreviations: CAI, complement activation index; BMI, body mass index; MAP, mean arterial pressure; UPRO, 24‑hour urine protein; eGFR, estimated glomerular filtration rate. M, E, S, T refer to the Oxford MEST scores. R values are Spearman's rank correlation coefficients. P values are two‑tailed and were not adjusted for multiple testing.

### Supplementary Table S2. Sensitivity Analyses of the Association Between Complement Activation Index (High vs. Low CAI) and the Composite Renal Outcome

| **Sensitivity Analysis** | **HR (95% CI)** | **P Value** |
| --- | --- | --- |
| **Primary analysis** |  |  |
| **Adjusted I model (age, sex, hypertension, diabetes)** | 4.57 (2.10–9.96) | <0.001 |
| **Adjusted II model (further adjusted for eGFR, UPRO, MEST, immunosuppression)** | 2.58 (1.15–5.80) | 0.022 |
| **Alternative exposure definition** |  |  |
| **CAI per 1‑point increase (continuous, Adjust II)** | 1.87 (1.10–3.20) | 0.021 |
| **Alternative CAI cutoff (score 0–2 vs. 3–4, Adjust II)** | 1.49 (0.65–3.42) | 0.349 |
| **Excluding early events** |  |  |
| **Landmark analysis (excluding events <6 months, Adjust II)** | 3.40 (1.33–8.67) | 0.010 |
| **Handling non‑proportional hazards** |  |  |
| **Stratified Cox model by sex (Adjusted II variables without sex)** | 3.36 (1.48–7.63) | 0.004 |
| **Additional adjustment for components** |  |  |
| **CAI adjusted for its individual components (uC3, sC3, iC3, iC1q) + clinical factors** | 2.12 (0.89–5.05) | 0.090 |

All models are Cox proportional hazards models. The primary Adjust II model served as the reference. Abbreviations: HR, hazard ratio; CI, confidence interval; eGFR, estimated glomerular filtration rate; UPRO, 24‑hour proteinuria; MEST, Oxford pathologic scores. The stratified model addressed mild violation of the proportional hazards assumption by sex. The alternative cutoff (0–2 vs. 3–4) was prespecified to test the robustness of the primary cutoff. Landmark analysis excluded patients with events occurring within the first 6 months to mitigate potential immortal.

### Supplementary Table S3. Benjamini–Hochberg false discovery rate (FDR) correction for baseline comparisons between Low- and High-CAI groups.

| **Variable** | **P value** | **Q value** | **Nominal significance** | **FDR significance** |
| --- | --- | --- | --- | --- |
| **Urinary C3, mg/L** | <0.001 | <0.001 | Yes | Yes |
| **24-hour urine protein, g/24h** | <0.001 | <0.001 | Yes | Yes |
| **eGFR, mL/min/1.73 m²** | <0.001 | <0.001 | Yes | Yes |
| **Cystatin C, mg/L** | <0.001 | <0.001 | Yes | Yes |
| **Serum albumin, g/L** | <0.001 | <0.001 | Yes | Yes |
| **Serum creatinine, mg/dL** | <0.001 | 0.001 | Yes | Yes |
| **Serum IgG, g/L** | <0.001 | 0.002 | Yes | Yes |
| **Glomerular C3 deposition** | <0.001 | 0.003 | Yes | Yes |
| **Urea nitrogen, mg/dL** | 0.001 | 0.005 | Yes | Yes |
| **Serum C3, g/L** | 0.001 | 0.005 | Yes | Yes |
| **Total cholesterol, mmol/L** | 0.003 | 0.014 | Yes | Yes |
| **Triglycerides, mmol/L** | 0.003 | 0.014 | Yes | Yes |
| **Lactate dehydrogenase, U/L** | 0.008 | 0.028 | Yes | Yes |
| **Oxford T score** | 0.009 | 0.031 | Yes | Yes |
| **Serum globulin, g/L** | 0.010 | 0.034 | Yes | Yes |
| **Oxford M1 lesion** | 0.026 | 0.080 | Yes | No |
| **ARB/ACEI use** | 0.029 | 0.084 | Yes | No |
| **LDL-C, mmol/L** | 0.036 | 0.098 | Yes | No |
| **CD20+ B cells, /μL** | 0.046 | 0.120 | Yes | No |
| **Hypertension** | 0.083 | 0.169 | No | No |
| **Platelets, ×10⁹/L** | 0.073 | 0.169 | No | No |
| **HDL-C, mmol/L** | 0.078 | 0.169 | No | No |
| **Glomerular C1q deposition** | 0.081 | 0.169 | No | No |
| **SGLT2 inhibitor use** | 0.081 | 0.169 | No | No |
| **Methylprednisolone pulse therapy** | 0.106 | 0.208 | No | No |
| **Mean arterial pressure, mmHg** | 0.112 | 0.210 | No | No |
| **Glomerular IgM deposition** | 0.116 | 0.210 | No | No |
| **Hemoglobin, g/L** | 0.154 | 0.252 | No | No |
| **Serum IgM, g/L** | 0.149 | 0.252 | No | No |
| **Cyclophosphamide use** | 0.151 | 0.252 | No | No |
| **Diabetes mellitus** | 0.229 | 0.363 | No | No |
| **Body mass index, kg/m²** | 0.374 | 0.572 | No | No |
| **CD4+ T cells, /μL** | 0.394 | 0.578 | No | No |
| **Oxford S1 lesion** | 0.401 | 0.578 | No | No |
| **Age, years** | 0.486 | 0.648 | No | No |
| **Serum C4, g/L** | 0.489 | 0.648 | No | No |
| **CD8+ T cells, /μL** | 0.487 | 0.648 | No | No |
| **Glomerular IgA deposition** | 0.774 | 0.998 | No | No |
| **Female sex** | 1.000 | 1.000 | No | No |
| **Serum IgA, g/L** | 0.925 | 1.000 | No | No |
| **Serum IgE, IU/mL** | 0.995 | 1.000 | No | No |
| **Oxford E1 lesion** | 0.865 | 1.000 | No | No |
| **Glomerular IgG deposition** | 0.899 | 1.000 | No | No |
| **Baseline CRRT** | 1.000 | 1.000 | No | No |
| **Prednisone use** | 1.000 | 1.000 | No | No |
| **NEFECON use** | 1.000 | 1.000 | No | No |
| **Mycophenolate mofetil use** | 0.870 | 1.000 | No | No |
| **Rituximab use** | 1.000 | 1.000 | No | No |
| **Other immunosuppressant use** | 1.000 | 1.000 | No | No |

P values from all 49 baseline comparisons between the Low- and High-CAI groups presented in Tables 1 and 2 were corrected using the Benjamini–Hochberg method. Continuous variables were compared using Wilcoxon rank-sum tests, and categorical variables using chi-square tests. Nominal significance was defined as P < 0.05, and FDR significance as Q < 0.05. Variables that were nominally significant but did not remain significant after FDR correction should be interpreted as exploratory. The composite renal endpoint was not included because it is a follow-up outcome rather than a baseline characteristic. Abbreviations: CAI, complement activation index; eGFR, estimated glomerular filtration rate; LDL-C, low-density lipoprotein cholesterol; HDL-C, high-density lipoprotein cholesterol; ARB/ACEI, angiotensin receptor blocker/angiotensin-converting enzyme inhibitor; SGLT2, sodium-glucose cotransporter 2; CRRT, continuous renal replacement therapy.
